# The impact of triglyceride-glucose index on the prognosis of post-PCI patients–a meta-analysis

**DOI:** 10.3389/fcvm.2024.1396865

**Published:** 2024-06-17

**Authors:** Yi-Fei Wang, Xiao-Han Kong, Hui-Min Tao, Li Tao

**Affiliations:** ^1^ Department of Cardiology, Nanjing First Hospital, Nanjing Medical University, Nanjing, China; ^2^ Department of Obstetrics and Gynecology, Jiangsu Women and Children Health Hospital, Nanjing Medical University, Nanjing, China; ^3^ Nanjing Medical University, Nanjing, China

**Keywords:** percutaneous coronary intervention (PCI), TyG index, insulin resistance, meta-analysis, coronary artery disease

## Abstract

**Background:**

Previous research has demonstrated the validity of the triglyceride-glucose (TyG) index as a robust measure of insulin resistance (IR) and its association with coronary artery disease (CAD). The objective of this study is to elucidate the relationship between the TyG index and the prognosis of patients underwent percutaneous coronary intervention (PCI) through a comprehensive systematic review and meta-analysis. Our goal is to provide a thorough analysis of the available evidence to offer more clarity on this association.

**Methods:**

A systematic and thorough search was carried out in the PubMed, Embase, Cochrane Library, and Web of Science databases, covering studies published in English from the beginning until October 1, 2023. The focus of the search was to gather relevant studies pertaining to the occurrence of major adverse cardiovascular events (MACE). To address the variability among the included studies, random or fixed effect models were utilized to summarize the hazard ratios (HR). In cases where heterogeneity was detected, subgroup or sensitivity analyses were performed to explore potential sources. To evaluate publication bias, the Egger or Begg test was employed.

**Results:**

This study incorporated a total of 17 studies. Individuals with the highest TyG index exhibited an elevated risk of major adverse cardiovascular events (MACEs) compared to those with the lowest TyG index (HR = 1.69; 95% CI: 1.47–1.95; *P* < 0.001). When analyzing the TyG index as a continuous variable, each standard deviation increase was associated with an HR of 1.60 (95% CI: 1.48–1.73; *P* < 0.001). Moreover, in patients diagnosed with acute coronary syndrome (ACS), higher TyG index levels showed a trend of increased risk of MACE (HR = 1.54; 95% CI: 1.27–1.86; *P* < 0.001). Furthermore, an elevated TyG index was found to be associated with a higher risk of in-stent restenosis (HR = 1.62; 95% CI: 1.29–2.03; *P* < 0.001), new-onset atrial fibrillation (HR = 2.97; 95% CI: 2.10–4.06; *P* = 0.014), and a reduction in quantitative flow ratio (HR = 1.35; 95% CI: 1.101–1.592; *P* = 0.005). Subgroup analysis indicated the risk of MACE was comparable between varied durations of follow-up (*P* = 0.11). Furthermore, regression analysis revealed that the positive association between TyG index and the risk of MACE did not differ between individuals with or without diabetes (*P* = 0.23).

**Conclusion:**

An increase in the TyG index may lead to a higher vulnerability to major adverse cardiovascular events (MACE) in patients underwent PCI and there was no significant difference in the risk of major adverse cardiovascular events (MACE) between diabetic and non-diabetic individuals.

## 1 Background

Coronary artery disease (CAD) is a highly prevalent cardiovascular condition that affects individuals worldwide. In the management of this condition, percutaneous coronary intervention (PCI) has emerged as a crucial treatment. Despite advancements in technology and the use of second-generation drug-eluting stents (DES), the occurrence of post-PCI adverse events continues to pose a significant public health concern. Specifically, the incidence of in-stent restenosis (ISR) and the need for target lesion revascularization (TLR) after PCI still increases with an annual growth rate of 1%–2% (1, 2).

Patients with diabetes mellitus (DM) have a high risk of cardiovascular diseases. Insulin resistance (IR) plays a significant role in the development of type 2 diabetes mellitus (T2DM). This condition also impacts individuals without diabetes (3, 4). Previous research has demonstrated that regardless of BMI, insulin resistance (IR) increases the susceptibility to cardiovascular adverse events (5, 6). The hypoglycemic-hyperinsulinemic clamp test, widely considered the gold standard for assessing IR, is, however, both burdensome and costly. In contrast, for many years, the triglyceride-glucose (TyG) index has been acknowledged as a dependable and economical substitute for diagnosing IR (7). Yet, additional investigation is needed to establish its predictive capability for cardiovascular outcomes.

Previous research has investigated the relationship between levels of TyG and the risk of coronary artery disease (CAD), and findings indicate that baseline TyG levels can independently predict the occurrence of CAD (8, 9). In a meta-analysis of 41 studies, Liang, Wang et al. discovered a strong association between TyG index levels and the risk, severity, and prognosis of CAD (10). Furthermore, for patients who underwent PCI (percutaneous coronary intervention), recent studies have identified the predictive value of TyG index for adverse cardiovascular events. Ma et al. specifically focused on patients with diabetes who underwent PCI and found a significant correlation between the TyG index and adverse cardiovascular events (11–27). Interestingly, Yang et al. reported that the TyG index was not an effective predictive factor for post-PCI adverse cardiovascular outcomes in non-diabetic patients (21). This systematic review and meta-analysis aim to assess the potential prognostic role of TyG index in patients undergoing PCl for stable or acute CAD.

## 2 Methods

The present report follows the established guidelines outlined by the Preferred Reporting Items for Systematic Reviews and Meta-Analyses (PRISMA) (28) and the Meta-analysis of Observational Studies in Epidemiology (MOOSE) (29). Furthermore, it has undergone registration for meta-analysis in PROSPERO, with the assigned registration number CRD42023467909.

### 2.1 Search strategy

To ensure the comprehensiveness of our study, we performed a thorough search in the following databases: PubMed, Embase, Cochrane Library, and Web of Science. The search encompassed the entire period from the establishment of these databases until October 1, 2023. Our search involved a mix of subject terms and free-text terms. The terms we used included “Coronary intervention”, “Percutaneous Coronary Intervention”, “PCI”, “coronary intervention angioplasty”, “Triglycerides”, “Triacylglycerol”, “triacyl glyceride”, and “TyG”. The specific methods for conducting the search are provided in Supplementary Material S1. Furthermore, we conducted a manual search of the references cited in published systematic reviews to ensure that we included all relevant literature.

### 2.2 Literature screening

The inclusion criteria for this study were as follows: (1) Participants: The study included patients who underwent percutaneous coronary intervention (PCI) for the treatment of coronary artery disease. (2) The exposure variable of interest was the TyG index, which was calculated using the formula Ln [fasting triglyceride (mg/dl) × fasting plasma glucose (mg/dl)/2]. (3) Study design: Only observational studies were considered for inclusion in this analysis. (4) The primary outcome measure was major adverse cardiovascular events (MACEs), which included all-cause death, cardiac death, myocardial infarction, revascularization, stroke, and heart failure. Secondary outcomes of interest included the occurrence of new-onset atrial fibrillation, in-stent restenosis (ISR), and reduction of quantitative flow ratio. (5) The included studies reported relative risks (RR), odds ratios (OR), and hazard ratios (HR) along with their corresponding 95% confidence intervals (CIs). Alternatively, studies provided raw data that could be used for the calculation of these effect measures.

The following studies were excluded: (1) The included sources will consist of reviews, case reports, research plans, and conference papers. (2) Clinical trials, animal or *in vitro* studies will be considered for inclusion. (3) Examples of publications that will be excluded are duplicate publications or studies with unavailable full texts. (4) Any literature that lacks information on outcome indicators will not be included in the analysis.

The literature screening process was carried out by two independent reviewers, HM TAO and L TAO. In case there were any differing opinions during the screening, the reviewers engaged in a constructive exchange of ideas to reach a consensus. Alternatively, if needed, a third reviewer named Wang was involved to provide a final decision.

### 2.3 Data extraction and quality assessment

HM TAO and L TAO, two independent reviewers, extracted information from the included literature. They focused on various details, including the first author, publication year, country, basic characteristics of the study population, exposure levels, follow-up duration, and outcome indicators. Their analysis covered a wide range of information.

The two independent reviewers, Wang and Kong, conducted the quality assessment of the included studies using the Newcastle-Ottawa Scale (NOS) (30). The assessment criteria for retrospective studies consist of eight aspects. These include the adequacy of case definition, representativeness of cases, selection of controls, definition of controls, comparability of cases and controls based on design or analysis, determination of the extent of exposure, the shared method for determining cases and controls, and comparison of non-response rates in cases and controls. For prospective studies, the evaluation encompasses several aspects as well. These include the representativeness of the exposure cohort, selection of the non-exposure cohort, confirmation of exposure, absence of the outcome of interest at the study's outset, comparability of cohorts through design or analysis, evaluation of outcomes, determination of whether the follow-up duration is sufficient to obtain outcomes, and adequacy of follow-up. Each aspect is assigned a score ranging from 0 to 1, except for comparability, which can receive up to 2 points. Therefore, a study is considered of high quality if it achieves a total score of 6 points or more out of 9.

### 2.4 Data integration and statistical analysis

We performed a meta-analysis using Stata 17.0 to examine a collection of diverse studies. To address inconsistencies in outcome measures, we used a conversion formula to transform odds ratios (OR) into risk ratios (RR), and aggregated RR and hazard ratios (HR) as effect sizes. To assess heterogeneity among studies, we employed the Q test and calculated the I^2^ statistic. For studies with no significant heterogeneity (I^2^ < 50% and *P* > 0.1), we applied the fixed-effects model (Mantel-Haenszel method) for the meta-analysis. Conversely, for studies exhibiting significant heterogeneity, we utilized the random-effects model (Der Simonian-Laird method). We further explored the extent and sources of heterogeneity through subgroup analyses and regression analyses, considering factors such as diabetes background and follow-up duration. Additionally, we conducted a sensitivity analysis to assess the robustness of the meta-analysis results. To evaluate publication bias in the included literature, we created a funnel plot and employed either Egger's or Begg's test for statistical assessment, making sure to have a minimum of 5 studies. If significant publication bias was found, we used the trim-and-fill method to estimate the potential impact on the findings.

## 3 Results

### 3.1 Literature screening results

**Figure 1:**
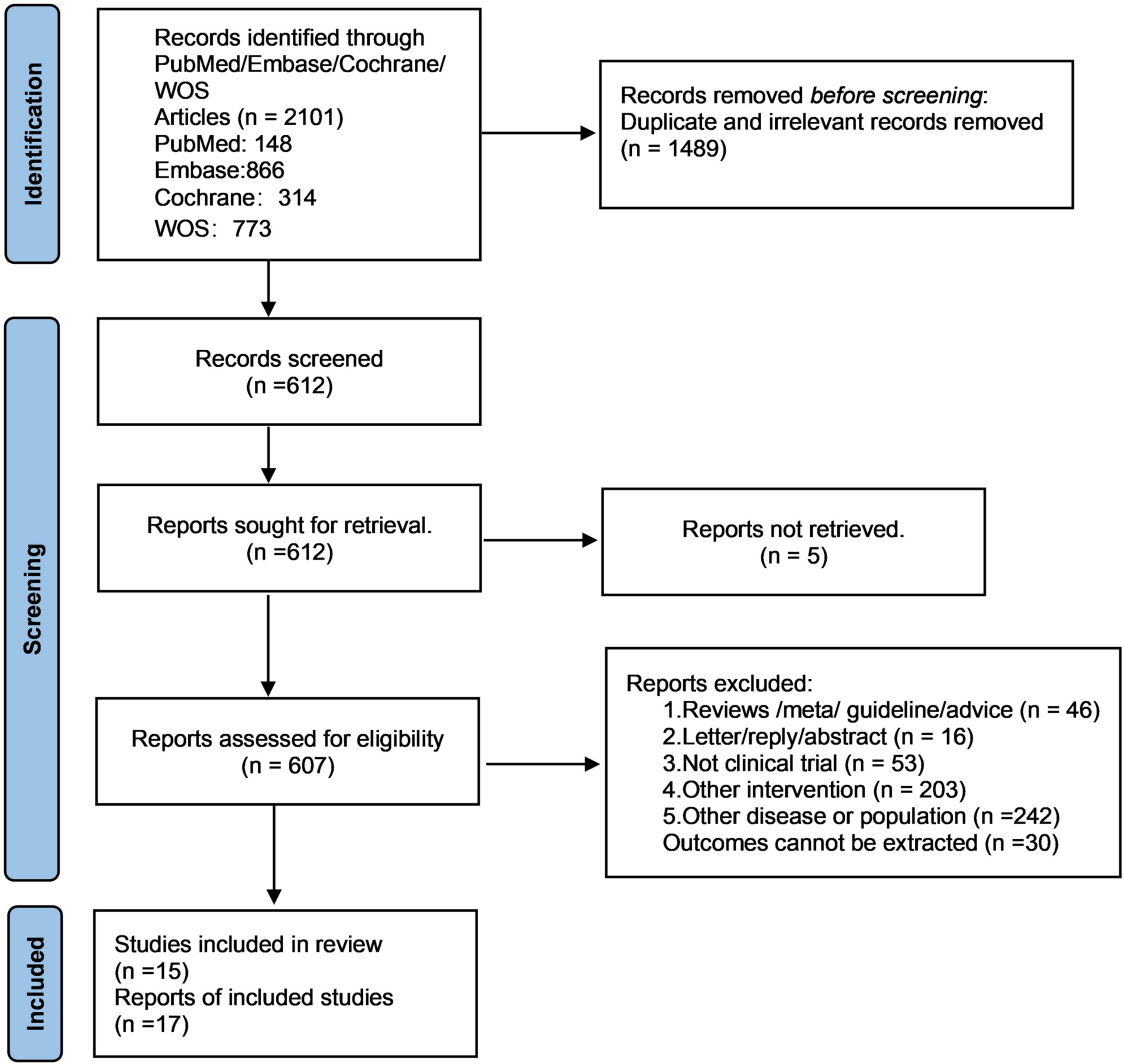
Flow chart of searching process for relevant studies.

After eliminating duplicate and irrelevant studies, a total of 2101 papers were obtained from the initial database search. The titles and abstracts of 612 articles were then examined. Ultimately, 17 studies (11–27) were included in this study. The search process for relevant studies is illustrated in Figure 1.

### 3.2 Basic characteristics of included studies

The studies included in the analysis involved a total of 22,467 participants, with 17,376 being males and 5,100 being females. The average age of the participants in these studies ranged from 58 to 66.6 years. The sample sizes of the studies varied, ranging from 214 cases to 5,489 cases. Ten studies explicitly mentioned that the patients included in their research underwent percutaneous coronary intervention (PCI) for acute coronary syndrome (ACS). Four studies focused on ISR, eleven studies examined the occurrence of post-PCI MACE, one study investigated new-onset atrial fibrillation, and one study explored the reduction in quantitative flow ratio. The follow-up duration ranged from 6 months to 5 years, with varying durations in different studies. Table 1 provides detailed information on the key characteristics of the studies included in this analysis.

**Table 1:** Characteristics of the studies included in the meta-analysis.

No.	Author	Year	Country	Total, *n*	Male, *n* (%)	Patients’ characteristics	Age	Hypertension, *n* (%)	Diabetes mellitus, *n* (%)	Influencing factors included in analysis
No. 1	Zhu	2021	China	1,574	1,218 (77.4)	Post-PCI patients with ACS	58.4 ± 9.4	1,016 (64.5)	544 (34.6)	Age, sex, BMI, LVEF, Hs-CRP, hypertension, diabetes mellitus, previous PCI, SYNTAX score, target vessel in LAD, target vessel in RCA, the application of intracoronary imagine, DES-sirolimus, total length of stents and minimal stent diameter
No. 2	Ferik	2022	Turkey	214	147 (68.8)	Post-PCI patients with CAD	64.0 ± 10.2	132 (61.6)	Non-diabetic patients	Gensini score,SYNTAX score,TGI,White blood cell count
No. 3	Guo	2023	China	1,414	1,103 (78)	Post-PCI patients with CCS	58 ± 9.33	906 (64)	597 (42.2)	Age, sex, BMI, previous PCI, presence of PAD, presence of multivessel CAD, high-sensitivity CRP, eGFR, presence of lesion's length ≥20 mm, stent length
No. 4	Huang	2022	China	922	734 (79.6)	CAD patients with MR who underwent PCI	64.1	552 (59.9)	–	Age, gender, smoking history, body mass index, left ventricular ejection fraction, hyperlipidemia, hypertension, diabetes mellitus, anemia, chronic kidney disease, acute myocardial infarction and atrial fibrillation, renin–angiotensin–aldosterone system inhibitor, beta-blockers, loop diuretics, mineralocorticoid receptor antagonist
No. 5	Kalyoncuoglu	2021	Turkey	124	94 (77.4)	Post-PCI patients with ACS	57 ± 9.1	74 (59.7)	37 (29.8)	Diabetes, CAD, Type of stent, TyG index
No. 6	Lin	2023	China	681	563 (82.7)	Post-PCI patients with T2DM diagnosed with CTO	59.16 ± 9.82	468 (68.7)	Patients with T2DM	TyG index, age, BMI, SBP, previous MI, previous PCI, TC, LDL-C, TG, FBG, HbA1c, eGFR, uric acid, and insulin
No. 7	Yang	2022	China	549	408 (80.4)	Post-PCI patients diagnosed with STEMI	63.0 (53.0–72.0)	269 (53)	154 (28)	Age, TyG index, eGFR
No. 8	Luo	2019	China	1,092	874 (80.0)	Post-PCI patients diagnosed with STEMI	62.3 ± 12.4	678 (62)	270 (24.7)	TyG index, Killip class >1, anaemia, albumin, uric acid, number of stents, LVEF
No. 9	Ma	2020	China	776	560 (72.1)	Post-PCI patients with T2DM diagnosed with ACS	61.2 ± 10	532 (68.5)	Patients with T2DM	TyG index tertiles, Age, BMI, DBP, HDL-C, Glycosylated haemoglobin, sex, Current smoking, Daily drinking, Previous MI, Past PCI, PAD, CKD, Cardiac failure, Insulin at discharge, Metformin at discharge, Alpha-glucosidase inhibitors at discharge, Sulfonylurea at discharge, Dipeptidyl peptidase 4 inhibitors at discharge, CAD severity
No. 10	Qin	2022	China	899	583 (64.8)	Post-PCI patients with T2DM diagnosed with ACS	59.8 ± 10.28	–	Patients with T2DM	Creatinine (mmol/L), WBC, Neut, Fibrinogen (g/L),LVEF (%),Duration of diabetes (years), GRACE risk score, TYG index
No. 11	Sun	2023	China	2,055	1,690 (82.2)	HF patients undergoing PCI	60.3 ± 11.0	1,186 (57.7)	792 (38.5)	Age, sex, heart rate, body mass index, NYHA class, prior PCI, platelet, albumin, TC, LDL-C, HDL-C, potassium, uric acid, LVEF, ARB, thiazide diuretics, spironolactone, sacubitril/valsartan, diffuse lesion, SYNTAX score, LM disease, in-stent restenosis, target vessel (LM), complete revascularization
No. 12	Wang	2022	China	1,694	1,339 (79.0)	ACS patients undergoing PCI	57.8 ± 9.7	1,025 (60.5)	618 (36.5)	Age sex hypertension, dyslipidemia, diabetes mellitus, prior MI, prior PCI, prior CABG, mean stent diameter, β-blocker, oral hypoglycemic agents, insulin, baseline LDL-C, baseline TC, baseline HDL-C, baseline HbA1C for baseline TyG index
No. 13	Xiong	2022	China	986	707 (71.7)	ACS patients undergoing PCI	66.6 ± 11.3	639 (64.8)	343 (34.7)	GRACE score, Female, bSS, ICR, LVEF, AMI, Diuretics
No. 14	Yang Jie	2021	China	5,489	4,358 (79.3)	Post-PCI patients without T2DM	57 ± 10.1	3,324 (60)	–	Previous stroke, SYNTAX score, and LVEF
No. 15	Yu	2022	China	241	203 (84.2)	STEMI patients undergoing PCI	62 ± 12	106 (44.0)	33 (13.7)	Age, sex, BMI, LVEF, smoking, hypertension, diabetes mellitus, previous myocardial infarction, creatine, culprit vessel, length of stents, in-stent minimal lumen diameter and in-stent diameter stenosis
No. 16	Zhang	2023	China	1,650	1,279 (77.5)	Post-PCI patients without T2DM	60.50 ± 10.19	886 (53.7)	–	STEMI, hyperlipidemia, Hb, and b-blocker medication at discharge
No. 17	Zhao	2021	China	2,107	1,516 (72.0)	ACS patients undergoing PCI	60.02 ± 9.03	1,305 (61.9)	721 (34.2)	Smoking history, hypertension, diabetes mellitus, previous MI, previous PCI, previous stroke, clinical diagnosis, TC, hs-CRP, eGFR, HbA1c, LVEF, ACEI/ARB at discharge, oral antidiabetic agents at discharge, insulin at discharge, LM disease, three-vessel disease, chronic total occlusion, SYNTAX score, complete revascularization, number of stents

AMI, acute myocardial infarction; ARB, angiotensin receptor blocker; BMI, body mass index; bSS, baseline SYNTAX score; CABG, coronary artery bypass graft; CAD, coronary artery disease; CRP, C-reactive protein; DBP, diastolic blood pressure; DES, drug-eluting stents; eGFR, estimated glomerular filtration rate; FBG, fasting blood glucose; Hb, Hemoglobin; HbA1C, Glycated Hemoglobin; HDL-C, high-density lipoprotein-cholesterol; Hs-CRP, high sensitivity-C reactive protein; ICR, incomplete revascularization; LAD, left anterior descending artery; LDL-C, low-density lipoprotein cholesterol; LM, left main artery; LVEF, left ventricular ejection fraction; MI, myocardial infarction; PAD, peripheral artery disease; PCI, percutaneous coronary intervention; RCA, right coronary artery; SBP, systolic blood pressure; TC, total cholesterol; TG, triglyceride; TGI, triglyceride glucose index; TyG, triglyceride glucose; WBC, white blood cell.

### 3.3 Quality assessment

Based on the NOS scale, the quality assessment reveals that 16 of the 17 studies included in the analysis obtained a score of 6 or above. For more detailed information can be found in Supplementary Table S2.

### 3.4 Meta-analysis results

#### 3.4.1 The link between TyG index and the risk of MACE after PCI

**Figure 2:**
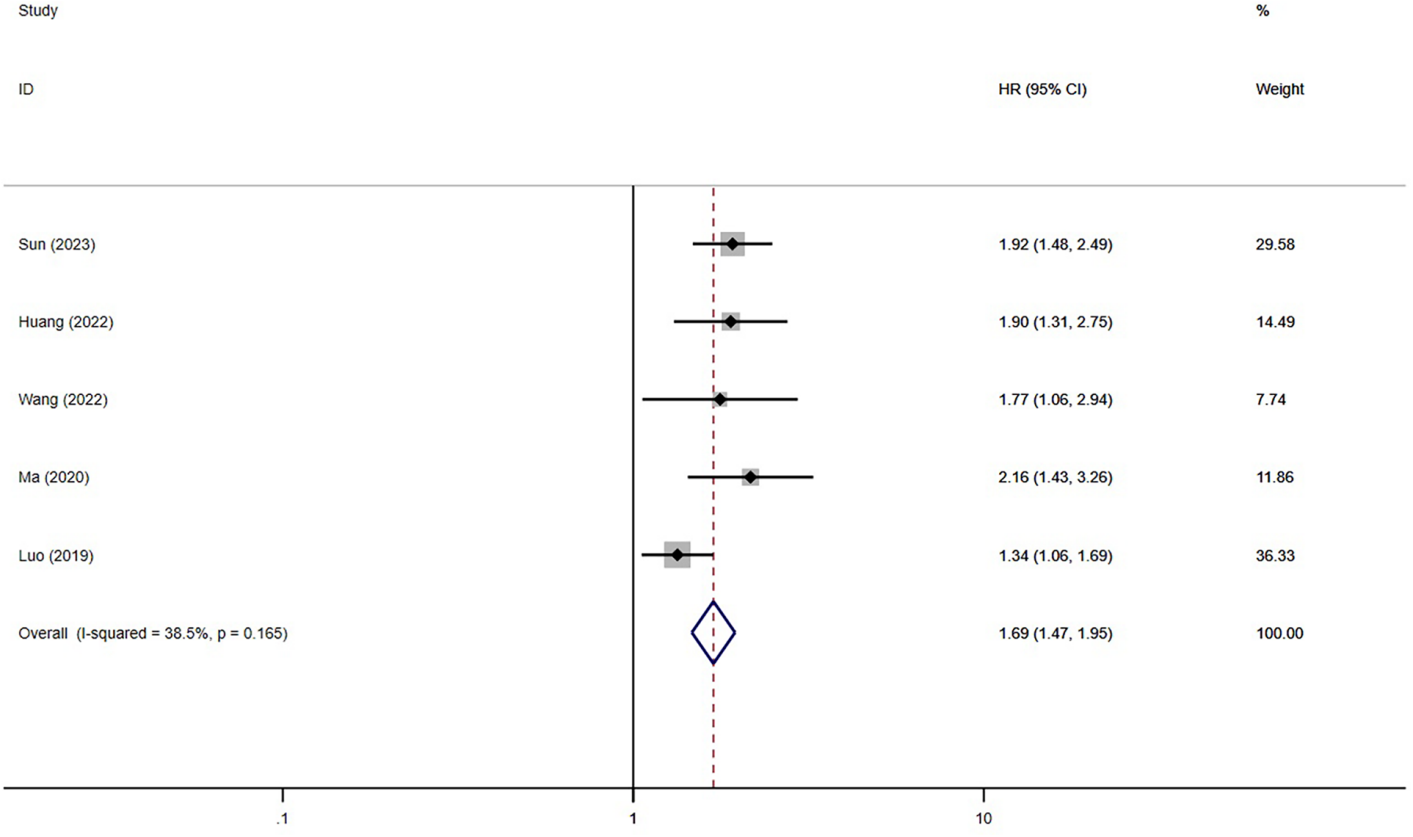
The meta-analysis of the incidence of MACE in post-PCI patients (categorized variables included).

**Figure 3:**
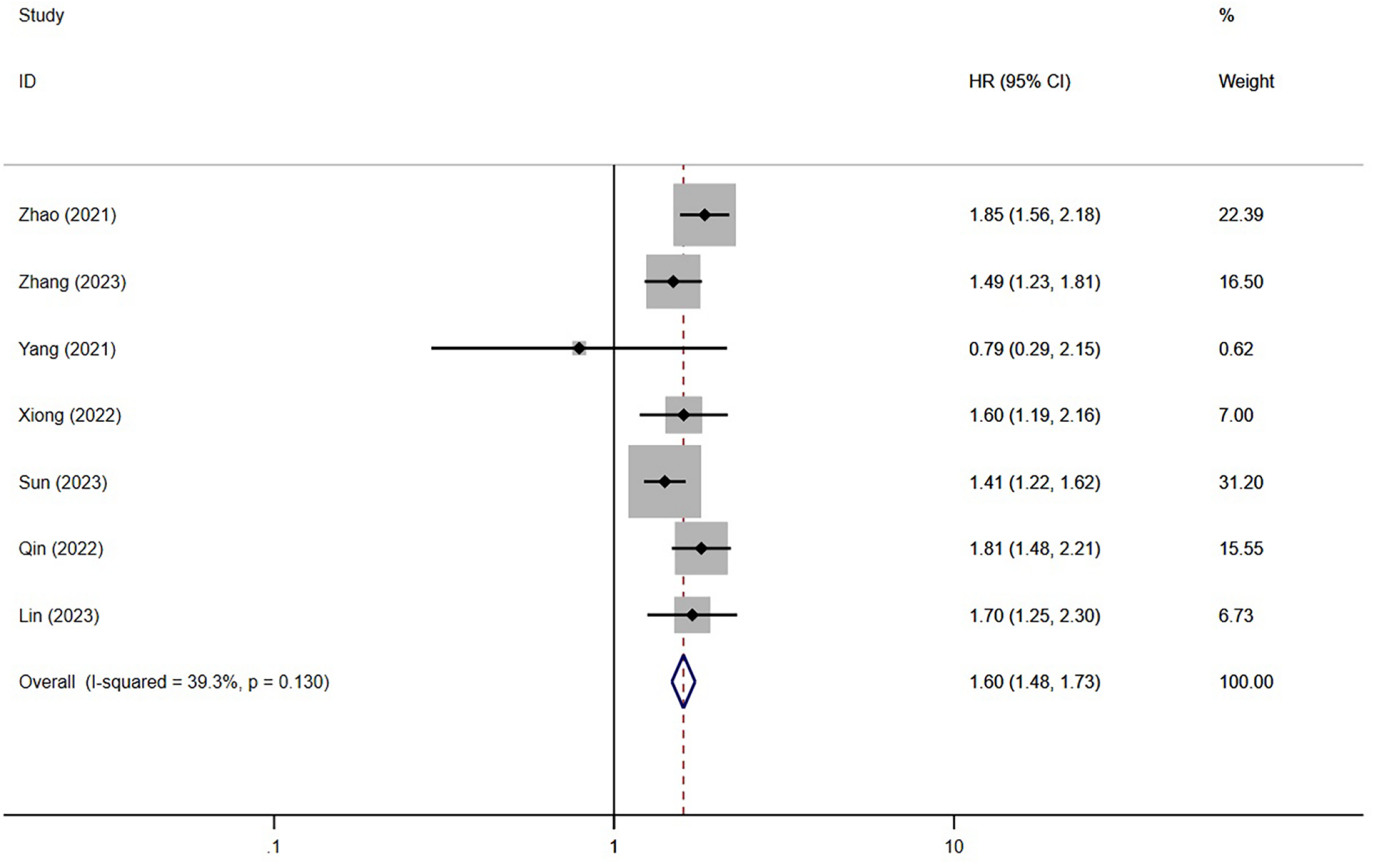
The meta-analysis of the incidence of MACE in post-PCI patients (continuous variables included).

A total of eleven studies have investigated the correlation between TyG index and the incidence of major adverse cardiovascular events (MACE) following percutaneous coronary intervention (PCI) (13, 15–21, 24–26) (Figures 2, 3). The researchers compared individuals categorized as having the highest TyG index to those with the lowest. Notably, the findings showed a significant elevation in the risk of MACE among individuals with the highest TyG index (HR = 1.69; 95% CI: 1.47–1.95; *P* < 0.001). When analyzed as a continuous variable, the HR for each SD increase in TyG index was 1.60 (95% CI: 1.48–1.73; *P* < 0.001) (Figure 3).

#### 3.4.2 The predictive value of TyG index for MACE in ACS patients underwent PCI

**Figure 4:**
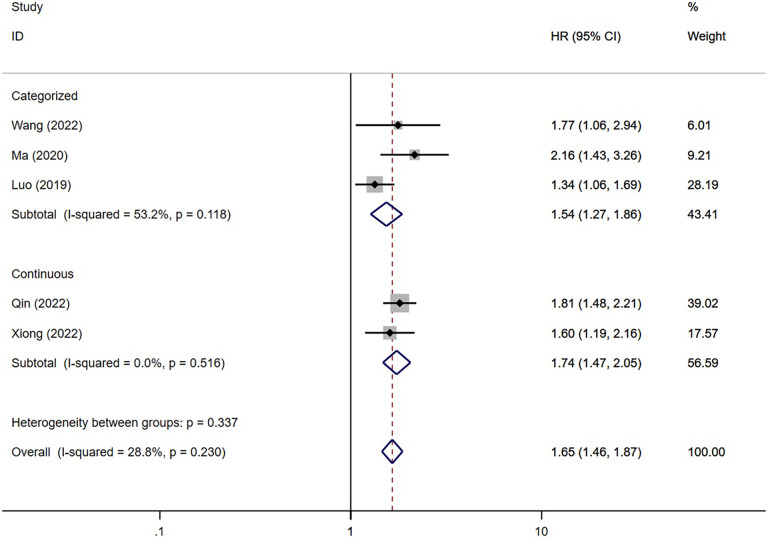
The meta-analysis of the incidence of MACE in post-PCI patients diagnosed with ACS.

The meta-analysis of five studies (16–18, 20, 26) revealed a significant association between TyG index and the risk of post-PCI MACE in patients diagnosed with ACS (*P* < 0.001). Patients with the highest TyG index had an increased risk of MACE compared to others (HR = 1.54; 95% CI: 1.27–1.86; *P* < 0.001). Furthermore, consistent findings were observed when analyzing the TyG index as a continuous variable in two studies (HR = 1.74; 95% CI: 1.47–2.05; *P* < 0.001) (Figure 4).

#### 3.4.3 The link between the TyG index and the occurrence of ISR

**Figure 5:**
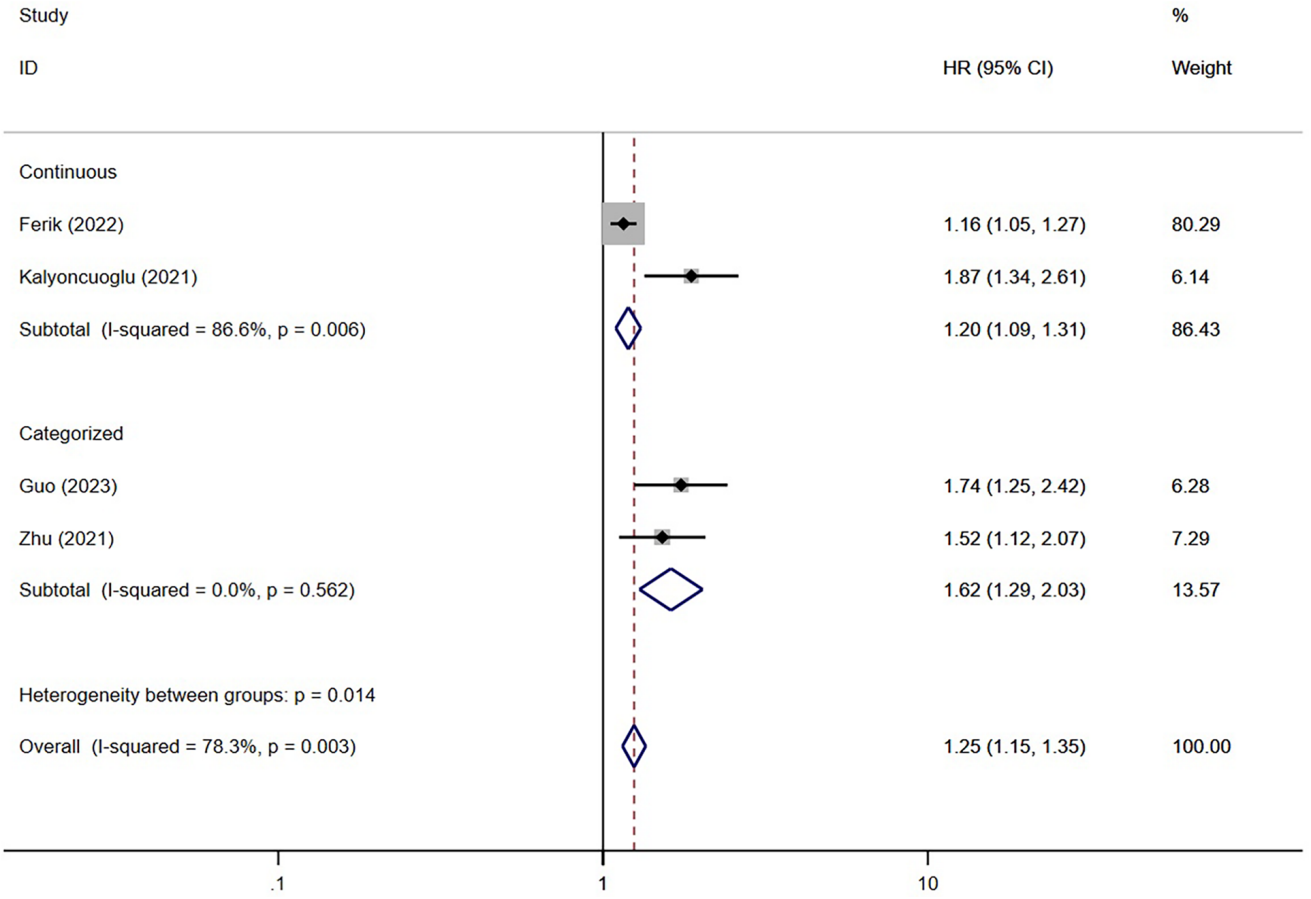
The meta-analysis of the incidence of ISR in post-PCI patients.

Four studies (11, 12, 14, 27) have examined the association between TyG and ISR. The findings of these studies suggested that the TyG index was associated with the risk of developing ISR significantly (HR = 1.25; 95% CI: 1.15–1.35; *P* < 0.001) (Figure 5).

#### 3.4.4 The link between the TyG index and the occurrence of other adverse events after PCI

Some articles among the included studies have explored the association between the TyG index and various cardiovascular adverse events. However, due to the limited number of studies (less than two), they could not be used for quantitative analysis. Through multivariate logistic regression analysis, a study conducted by Yang et al. discovered that the TyG index independently predicted the occurrence of new-onset atrial fibrillation (HR = 2.97; 95% CI: 2.10–4.06; *P* = 0.014) (22). In another study by Yu et al, a correlation was observed between the TyG index and a decrease in quantitative flow ratio (HR = 1.35; 95% CI: 1.101–1.592; *P* = 0.005) (23).

#### 3.4.5 Subgroup analysis and regression analysis

**Figure 6:**
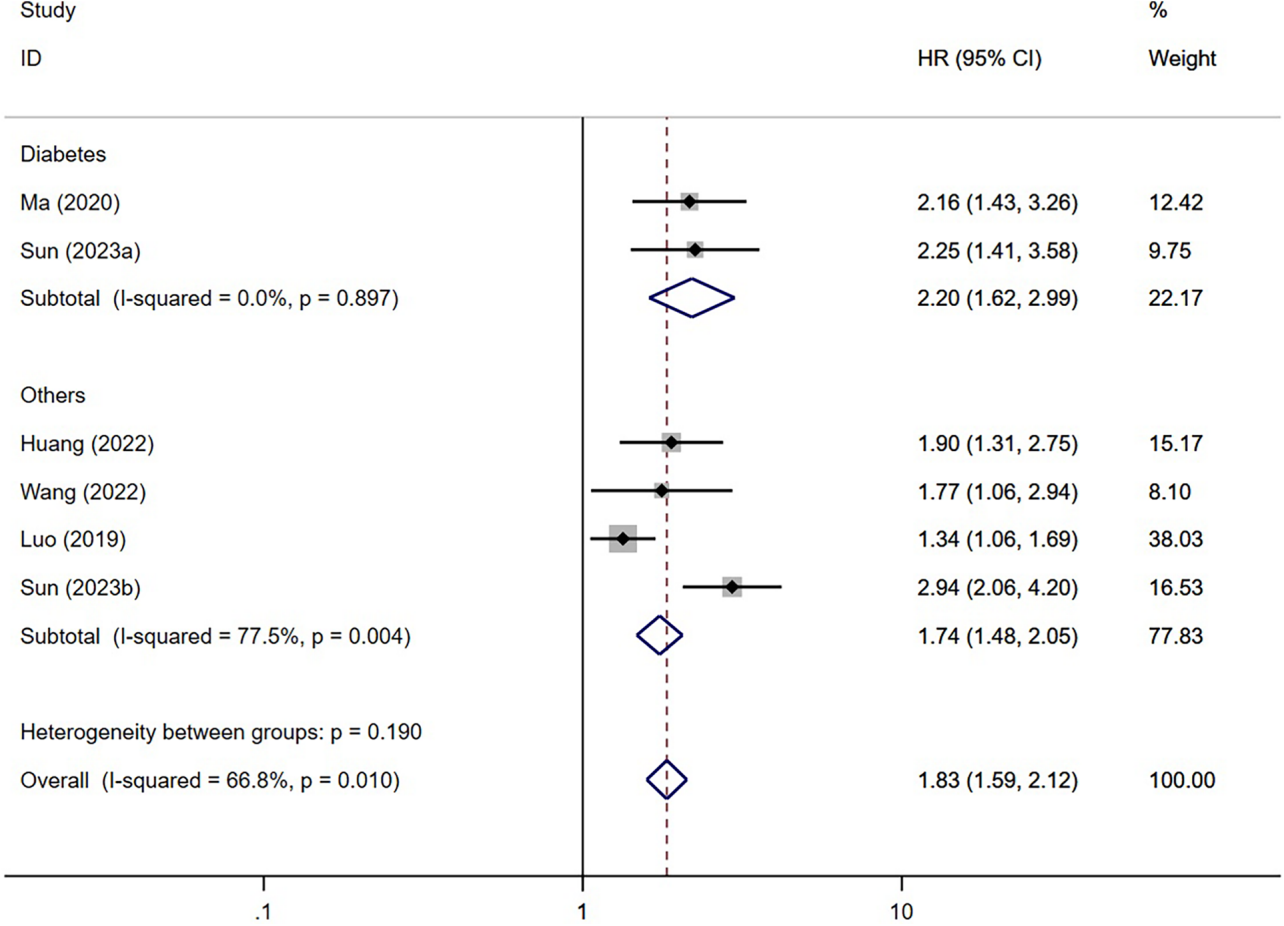
Subgroup analysis based on diabetes (categorized variables included. Sun (2023a), risk of MACE in patients with DM; Sun (2023b), risk of MACE in patients without DM).

**Figure 7:**
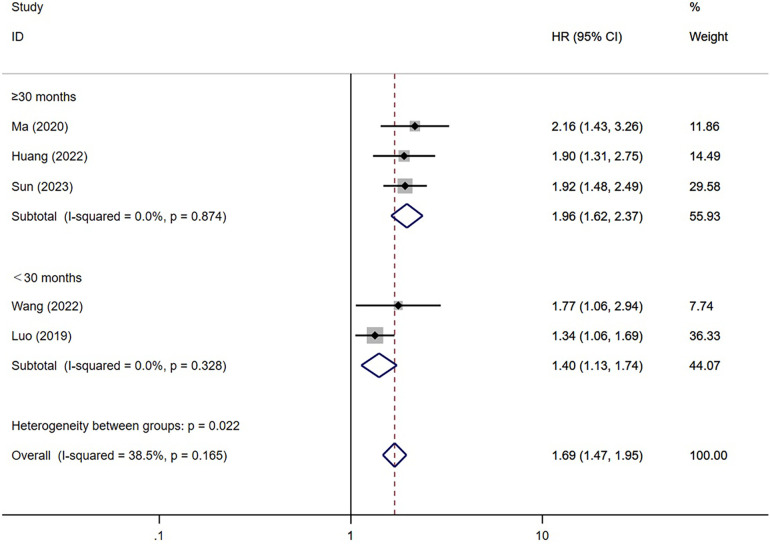
Subgroup analysis based on duration of follow-up (categorized variables included).

We performed a subgroup analysis of MACE based on diabetes and the duration of follow-up to explore the source of heterogeneity (Figures 6, 7). Considering diabetes status, a stratified analysis revealed that diabetic patients faced an elevated risk of MACE by a factor of 2.20 when exhibiting higher TyG index values instead of lower ones (95% CI: 1.62–2.99; *P* < 0.001). In alignment with this, those without diabetes also displayed a 1.74-fold increased risk of MACE under the same conditions (95% CI: 1.48–2.05; *P* < 0.001). Furthermore, a regression analysis demonstrated no significant variation in MACE risk among individuals irrespective of their diabetic status (*P* = 0.23). Furthermore, subgroup analysis was conducted based on the duration of follow-up. We categorized the follow-up time into two groups: those with a follow-up time of ≥30 months and those with a follow-up time of <30 months. The results indicated a significant association between an increase in the TyG index and a higher risk of MACE in both follow-up time categories [≥30 months (ES = 1.96; 95% CI: 1.62–2.37; *P* < 0.001) and <30 months (ES = 1.40; 95% CI: 1.13–1.74; *P* = 0.002)]. Regression analysis further revealed a stronger association between elevated TyG index levels and increased risk of MACE in the subgroup with longer follow-up time (*P* = 0.11).

### 3.5 Sensitivity analysis

To assess the influence of each study on the aggregated results, we conducted a sensitivity analysis using a leave-one-out method. Our primary outcome, MACE, was the focus of this analysis. Interestingly, none of the individual studies exerted a significant influence on the overall findings. This finding further supports the reliability of the overall results obtained from this meta-analysis.

### 3.6 Publication bias

To ensure the validity of the results obtained from the meta-analysis, we employed a variety of techniques. These included the utilization of a funnel plot, as well as the implementation of Egger's and Begg's tests, to identify any possible publication bias pertaining to Major Adverse Cardiovascular Events (MACE). The results obtained from these analyses revealed no significant evidence of publication bias for MACE.

## 4 Discussion

### 4.1 Main findings

Our research has demonstrated the significant value of the TyG index in predicting the risk of major adverse cardiovascular events (MACE) in patients who have undergone percutaneous coronary intervention (PCI). This finding is consistent with previous studies that have investigated the relationship between the TyG index and coronary artery disease (CAD), including its association with subclinical atherosclerosis, hypertension, coronary artery calcification, and arterial stiffness (8, 20, 31, 32). While PCI involves procedures such as stent implantation or balloon dilation, which improve blood flow and clinical outcomes, it is essential to also consider the occurrence of post-PCI cardiovascular adverse events. The heightened risk of MACE observed in patients with elevated TyG index levels could be attributed to insulin resistance (IR) (33, 34). Insulin resistance and disturbances in glucose metabolism contribute to oxidative stress, inflammation, and dysfunctional immune regulation, thereby exacerbating the development of arteriosclerosis and promoting plaque formation. Inflammation, triggered by insulin resistance and subsequent disturbances in glucose metabolism, may also play a role in the occurrence of in-stent restenosis (ISR), a common complication following stent implantation (35, 36). Additionally, the TyG index has demonstrated varying predictive abilities for different cardiovascular adverse events (19). This variation may provide valuable insights into the underlying mechanisms that contribute to its predictive ability.

Previous studies have demonstrated that the TyG index was associated with the prognosis of patients diagnosed with ACS or chronic coronary syndrome (CCS) (10). However, in response to an acute syndrome, patients with ACS may experience abnormal blood glucose levels due to stress-induced hyperglycemia (SIH) (37). SIH could result in the misbalance of oxidative equilibrium, trigger inflammation, and cause endothelial dysfunction, which can further worsen the prognosis (38). Additionally, the fluctuations in fasting blood glucose (FBG) levels can affect the calculation of the TyG index. Although researchers have recognized SIH as a potential confounding factor that may impact the predictive ability of the TyG index, there is currently a lack of relevant studies on this topic (39). In this study, we found a significant association between the TyG index and the risk of MACE in patients diagnosed with ACS. Further research is needed to investigate the predictive ability of the TyG index for eliminating factors with collinearity, such as SIH. Furthermore, subgroup analysis revealed that the predictive ability of the TyG index was comparable between patients with and without diabetes. This could be because both groups, diabetic and non-diabetic, share certain risk factors for insulin resistance (18).

Previous research has highlighted a limitation in using the TyG index at a specific time to accurately predict disease progression. This limitation arises from the variability of triglycerides and glucose levels (40). For instance, the fluctuation of glucose levels caused by stress-induced hyperglycemia (SIH) can impact the TyG index and introduce a confounding factor when assessing a patient's metabolic status (39, 41). Currently, some studies suggest the importance of dynamic monitoring of the TyG index or the use of cumulative TyG index. These approaches have been found to have greater clinical predictive value in the prognosis of coronary artery disease (20, 42).

Furthermore, subgroup analysis based on the duration of follow-up was conducted for the consideration of various factors such as endothelial dysfunction, smooth muscle cell proliferation, and the infiltration of inflammatory cells, all of which result from prolonged insulin resistance. Results have revealed that the predictive ability of TyG index was comparable between patients with varying durations of follow-up. Most studies had similar duration of follow-up, one research conducted by Luo et al., have reported the occurrence of MACE at different time points after PCI, but they did not analyze the change in the predictive ability of the TyG index based on different follow-up durations (16). Considering the long-term damage on cardiac function caused by insulin resistance and insufficient insulin signaling, it is essential for future research to explore the utility of dynamic monitoring of the TyG index or cumulative TyG index in patients who have undergone PCI. Additionally, it is necessary to clarify the time-dependence variation curve of the predictive value of the TyG index.

The predictive accuracy of the TyG index for CAD has been demonstrated to be higher compared to other indicators such as triglyceride (TG), atherogenic index of plasma (AIP), and the lipoprotein combine index (LCI) (6). Moreover, integrating the TyG index into the GRACE risk score has been found to enhance the prognostic predictive capability for patients who underwent PCI (18, 43) according to previous research. While additional studies are required, our research has contributed to the understanding of the predictive value of the TyG index and its potential utility in managing the prognosis of patients who underwent PCI.

### 4.2 Limitations

Our meta-analysis conducted a comprehensive analysis of the studies regarding the correlation between the TyG index and the prognosis of post-PCI patients. The findings of this study provide a solid evidence-based foundation in the field of medicine, which holds great potential in enhancing the postoperative conditions of PCI patients. However, it is important to acknowledge the existence of certain limitations in this study. Firstly, the HR and RR values were extracted from various multivariate analyses to account for the confounding factors present in each study. However, it should be noted that the confounding factors were not entirely consistent across all included studies. Secondly, most of the studies included in this meta-analysis predominantly involved Asian individuals. It is plausible that the pathogenesis of those undergoing PCI in different regions may exhibit variations, thus impacting the generalizability of our results. Nonetheless, our findings suggest a clinical value of the TyG index in predicting the prognosis of post-PCI patients.

## 5 Conclusion

An increase in the TyG index may lead to a higher vulnerability to major adverse cardiovascular events (MACE) in patients underwent PCI and there was no significant difference in the risk of major adverse cardiovascular events (MACE) between diabetic and non-diabetic individuals.

## Data Availability

The original contributions presented in the study are included in the article/**Supplementary Material**, further inquiries can be directed to the corresponding author.

## References

[1] Giustino G Colombo A Camaj A Yasumura K Mehran R Stone GW Coronary in-stent restenosis: JACC state-of-the-art review. J Am Coll Cardiol. (2022) 80(4):348–72. 10.1016/j.jacc.2022.05.017 35863852

[2] Bittl JA Baber U Bradley SM Wijeysundera DN. Duration of dual antiplatelet therapy: a systematic review for the 2016 ACC/AHA guideline focused update on duration of dual antiplatelet therapy in patients with coronary artery disease: a report of the American college of cardiology/American heart association task force on clinical practice guidelines. J Am Coll Cardiol. (2016) 68(10):1116–39. 10.1016/j.jacc.2016.03.512 27036919

[3] Gast KB Tjeerdema N Stijnen T Smit JW Dekkers OM. Insulin resistance and risk of incident cardiovascular events in adults without diabetes: meta-analysis. PLoS One. (2012) 7(12):e52036. 10.1371/journal.pone.0052036 23300589 PMC3532497

[4] Ormazabal V Nair S Elfeky O Aguayo C Salomon C Zuñiga FA. Association between insulin resistance and the development of cardiovascular disease. Cardiovasc Diabetol. (2018) 17(1):122. 10.1186/s12933-018-0762-4 30170598 PMC6119242

[5] Mechanick JI Farkouh ME Newman JD Garvey WT. Cardiometabolic-based chronic disease, adiposity and dysglycemia drivers: JACC state-of-the-art review. J Am Coll Cardiol. (2020) 75(5):525–38. 10.1016/j.jacc.2019.11.044 32029136 PMC7187687

[6] Sánchez-Íñigo L Navarro-González D Fernández-Montero A Pastrana-Delgado J Martínez JA. The TyG index may predict the development of cardiovascular events. Eur J Clin Invest. (2016) 46(2):189–97. 10.1111/eci.12583 26683265

[7] Satilmisoglu MH Ozyilmaz SO Gul M Ak Yildirim H Kayapinar O Gokturk K Predictive values of D-dimer assay, GRACE scores and TIMI scores for adverse outcome in patients with non-ST-segment elevation myocardial infarction. Ther Clin Risk Manag. (2017) 13:393–400. 10.2147/TCRM.S124794 28408834 PMC5384739

[8] Ding X Wang X Wu J Zhang M Cui M. Triglyceride-glucose index and the incidence of atherosclerotic cardiovascular diseases: a meta-analysis of cohort studies. Cardiovasc Diabetol. (2021) 20(1):76. 10.1186/s12933-021-01268-9 33812373 PMC8019501

[9] Zhao J Fan H Wang T Yu B Mao S Wang X Tyg index is positively associated with risk of CHD and coronary atherosclerosis severity among NAFLD patients. Cardiovasc Diabetol. (2022) 21(1):123. 10.1186/s12933-022-01548-y 35778734 PMC9250269

[10] Liang S Wang C Zhang J Liu Z Bai Y Chen Z Triglyceride-glucose index and coronary artery disease: a systematic review and meta-analysis of risk, severity, and prognosis. Cardiovasc Diabetol. (2023) 22(1):170. 10.1186/s12933-023-01906-4 37415168 PMC10327356

[11] Ferik ÖK Sayın BY Akbuğa K Zorlu Ç. Association between insulin resistance estimated by triglyceride glucose Index and in-stent restenosis in non-diabetic patients. EJCM. (2022) 10(1):12–7. 10.32596/ejcm.galenos.2022.2021-11-060

[12] Guo X Shen R Yan S Su Y Ma L. Triglyceride-glucose index for predicting repeat revascularization and in-stent restenosis in patients with chronic coronary syndrome undergoing percutaneous coronary intervention. Cardiovasc Diabetol. (2023) 22(1):43. 10.1186/s12933-023-01779-7 36864455 PMC9983161

[13] Huang H Li Q Liu J Qiao L Chen S Lai W Association between triglyceride glucose index and worsening heart failure in significant secondary mitral regurgitation following percutaneous coronary intervention. Cardiovasc Diabetol. (2022) 21(1):260. 10.1186/s12933-022-01680-9 36443743 PMC9706938

[14] Kalyoncuoglu M Ozkan AA Kaya A Yüksel Y Dogan N Gurmen AT. A new predictor of in-stent restenosis in patients undergoing elective percutaneous coronary İntervention: triglyceride glucose index. Int J Cardiovasc Acad. (2021) 7(2):50–4. 10.4103/ijca.ijca_15_21

[15] Lin XL Li QY Zhao DH Liu JH Fan Q. A high triglyceride-glucose index associated with adverse cardiovascular events in patients with type 2 diabetes mellitus and chronic total occlusion after percutaneous coronary intervention. J Investig Med. (2023) 71(5):471–81. 10.1177/10815589231152823 36727463

[16] Luo E Wang D Yan G Qiao Y Liu B Hou J High triglyceride-glucose index is associated with poor prognosis in patients with acute ST-elevation myocardial infarction after percutaneous coronary intervention. Cardiovasc Diabetol. (2019) 18(1):150. 10.1186/s12933-019-0957-3 31722708 PMC6852896

[17] Ma X Dong L Shao Q Cheng Y Lv S Sun Y Triglyceride glucose index for predicting cardiovascular outcomes after percutaneous coronary intervention in patients with type 2 diabetes mellitus and acute coronary syndrome. Cardiovasc Diabetol. (2020) 19(1):31. 10.1186/s12933-020-01006-7 32156279 PMC7063826

[18] Qin Z Xu S Yuan R Wang Z Lu Y Xu Y Combination of TyG index and GRACE risk score as long-term prognostic marker in patients with ACS complicated with T2DM undergoing PCI. Diabetes Metab Syndr Obes. (2022) 15:3015–25. 10.2147/DMSO.S376178 36196143 PMC9527003

[19] Sun T Huang X Zhang B Ma M Chen Z Zhao Z Prognostic significance of the triglyceride-glucose index for patients with ischemic heart failure after percutaneous coronary intervention. Front Endocrinol (Lausanne). (2023) 14:1100399. 10.3389/fendo.2023.1100399 36814584 PMC9939475

[20] Wang Y Wang Y Sun S Liu X Zhao W Li W Triglyceride-glucose index level and variability and outcomes in patients with acute coronary syndrome undergoing percutaneous coronary intervention: an observational cohort study. Lipids Health Dis. (2022) 21(1):134. 10.1186/s12944-022-01731-w 36482415 PMC9733246

[21] Yang J Tang YD Zheng Y Li C Zhou Q Gao J The impact of the triglyceride-glucose index on poor prognosis in nondiabetic patients undergoing percutaneous coronary intervention. Front Endocrinol (Lausanne). (2021) 12:710240. 10.3389/fendo.2021.710240 34489866 PMC8417234

[22] Ling Y Fu C Fan Q Liu J Jiang L Tang S. Triglyceride-glucose index and new-onset atrial fibrillation in ST-segment elevation myocardial infarction patients after percutaneous coronary intervention. Front Cardiovasc Med. (2022) 9:838761. 10.3389/fcvm.2022.838761 35345486 PMC8957253

[23] Yu B Mo Y Hu X Wang W Liu J Jin J Triglyceride-glucose index is associated with quantitative flow ratio in patients with acute ST-elevation myocardial infarction after percutaneous coronary intervention. Front Cardiovasc Med. (2022) 9:1002030. 10.3389/fcvm.2022.1002030 36158820 PMC9493184

[24] Zhang Y Chu C Zhong Z Luo YB Ning FF Guo N. High triglyceride-glucose index is associated with poor cardiovascular outcomes in Chinese acute coronary syndrome patients without diabetes mellitus who underwent emergency percutaneous coronary intervention with drug-eluting stents. Front Endocrinol (Lausanne). (2023) 14:1101952. 10.3389/fendo.2023.1101952 36875470 PMC9975349

[25] Zhao Q Cheng YJ Xu YK Zhao ZW Liu C Sun TN Comparison of various insulin resistance surrogates on prognostic prediction and stratification following percutaneous coronary intervention in patients with and without type 2 diabetes mellitus. Cardiovasc Diabetol. (2021) 20(1):190. 10.1186/s12933-021-01383-7 34537077 PMC8449896

[26] Xiong S Chen Q Chen X Hou J Chen Y Long Y Adjustment of the GRACE score by the triglyceride glucose index improves the prediction of clinical outcomes in patients with acute coronary syndrome undergoing percutaneous coronary intervention. Cardiovasc Diabetol. (2022) 21(1):145. 10.1186/s12933-022-01582-w 35932019 PMC9356419

[27] Zhu Y Liu K Chen M Liu Y Gao A Hu C Triglyceride-glucose index is associated with in-stent restenosis in patients with acute coronary syndrome after percutaneous coronary intervention with drug-eluting stents. Cardiovasc Diabetol. (2021) 20(1):137. 10.1186/s12933-021-01332-4 34238294 PMC8268452

[28] Hutton B Salanti G Caldwell DM Chaimani A Schmid CH Cameron C The PRISMA extension statement for reporting of systematic reviews incorporating network meta-analyses of health care interventions: checklist and explanations. Ann Intern Med. (2015) 162(11):777–84. 10.7326/M14-2385 26030634

[29] Stroup DF Berlin JA Morton SC Olkin I Williamson GD Rennie D Meta-analysis of observational studies in epidemiology: a proposal for reporting. Meta-analysis of observational studies in epidemiology (MOOSE) group. JAMA. (2000) 283(15):2008–12. 10.1001/jama.283.15.2008 10789670

[30] Stang A. Critical evaluation of the Newcastle-Ottawa scale for the assessment of the quality of nonrandomized studies in meta-analyses. Eur J Epidemiol. (2010) 25(9):603–5. 10.1007/s10654-010-9491-z 20652370

[31] Wang X Xu W Song Q Zhao Z Meng X Xia C Association between the triglyceride-glucose index and severity of coronary artery disease. Cardiovasc Diabetol. (2022) 21(1):168. 10.1186/s12933-022-01606-5 36050734 PMC9438180

[32] Yan Y Wang D Sun Y Ma Q Wang K Liao Y Triglyceride-glucose index trajectory and arterial stiffness: results from Hanzhong adolescent hypertension cohort study. Cardiovasc Diabetol. (2022) 21(1):33. 10.1186/s12933-022-01453-4 35216614 PMC8876112

[33] Park K Ahn CW Lee SB Kang S Nam JS Lee BK Elevated TyG index predicts progression of coronary artery calcification. Diabetes Care. (2019) 42(8):1569–73. 10.2337/dc18-1920 31182490

[34] Brito ADM Hermsdorff HHM Filgueiras MS Suhett LG Vieira-Ribeiro SA Franceschini S Predictive capacity of triglyceride-glucose (TyG) index for insulin resistance and cardiometabolic risk in children and adolescents: a systematic review. Crit Rev Food Sci Nutr. (2021) 61(16):2783–92. 10.1080/10408398.2020.1788501 32744083

[35] Piatti P Di Mario C Monti LD Fragasso G Sgura F Caumo A Association of insulin resistance, hyperleptinemia, and impaired nitric oxide release with in-stent restenosis in patients undergoing coronary stenting. Circulation. (2003) 108(17):2074–81. 10.1161/01.CIR.0000095272.67948.17 14530196

[36] Tao LC Xu JN Wang TT Hua F Li JJ. Triglyceride-glucose index as a marker in cardiovascular diseases: landscape and limitations. Cardiovasc Diabetol. (2022) 21(1):68. 10.1186/s12933-022-01511-x 35524263 PMC9078015

[37] Karakasis P Stalikas N Patoulias D Pamporis K Karagiannidis E Sagris M Prognostic value of stress hyperglycemia ratio in patients with acute myocardial infarction: a systematic review with Bayesian and frequentist meta-analysis. Trends Cardiovasc Med. (2023) 30:S1050–1738(23)00107-X. 10.1016/j.tcm.2023.11.006 38042441

[38] Mapanga RF Joseph D Symington B Garson KL Kimar C Kelly-Laubscher R Detrimental effects of acute hyperglycaemia on the rat heart. Acta Physiol (Oxf). (2014) 210(3):546–64. 10.1111/apha.12184 24286628

[39] Al Jumaily T Rose'Meyer RB Sweeny A Jayasinghe R. Cardiac damage associated with stress hyperglycaemia and acute coronary syndrome changes according to level of presenting blood glucose. Int J Cardiol. (2015) 196:16–21. 10.1016/j.ijcard.2015.05.143 26070178

[40] Alizargar J Bai CH Hsieh NC Wu SV. Use of the triglyceride-glucose index (TyG) in cardiovascular disease patients. Cardiovasc Diabetol. (2020) 19(1):8. 10.1186/s12933-019-0982-2 31941513 PMC6963998

[41] Ayhan H Durmaz T Keleş T Bayram NA Bilen E Akçay M The relationship between acute coronary syndrome and stress hyperglycemia. Exp Clin Endocrinol Diabetes. (2014) 122(4):222–6. 10.1055/s-0034-1367002 24771010

[42] Cui H Liu Q Wu Y Cao L. Cumulative triglyceride-glucose index is a risk for CVD: a prospective cohort study. Cardiovasc Diabetol. (2022) 21(1):22. 10.1186/s12933-022-01456-1 35144621 PMC8830002

[43] Pang S Miao G Zhou Y Du Y Rui Z Zhao X. Addition of TyG index to the GRACE score improves prediction of adverse cardiovascular outcomes in patients with non-ST-segment elevation acute coronary syndrome undergoing percutaneous coronary intervention: a retrospective study. Front Cardiovasc Med. (2022) 9:957626. 10.3389/fcvm.2022.957626 36093151 PMC9453480

