# Supplementary material for: The impact of triglyceride-glucose index on the prognosis of post-PCI patients–a meta-analysis
Source: Front Cardiovasc Med. 2024 Jun 17;11:1396865. doi: 10.3389/fcvm.2024.1396865 (PMC11216054; doi:10.3389/fcvm.2024.1396865)

# Literature search strategy

**1.Pubmed**

| Search number | Query |
| --- | --- |
| #1 | "Percutaneous Coronary Intervention"[Mesh] |
| #2 | ((((Percutaneous Coronary Intervention[Title/Abstract]) OR (PCI[Title/Abstract])) OR (coronary intervention[Title/Abstract])) OR (angioplasty[Title/Abstract])) OR (stent*[Title/Abstract]) |
| #3 | ("Percutaneous Coronary Intervention"[Mesh]) OR (((((Percutaneous Coronary Intervention[Title/Abstract]) OR (PCI[Title/Abstract])) OR (coronary intervention[Title/Abstract])) OR (angioplasty[Title/Abstract])) OR (stent*[Title/Abstract])) |
| #4 | ((((((Triglycerides[MeSH Major Topic]) OR (Triglycerides[Title/Abstract])) OR (Triacylglycerol[Title/Abstract])) OR (Triacylglycerols[Title/Abstract])) OR (Triglyceride[Title/Abstract])) OR (tryglyceride[Title/Abstract])) OR (triacyl glyceride[Title/Abstract]) |
| #5 | "Blood Glucose"[Mesh] |
| #6 | (((((Blood Glucose[Title/Abstract]) OR (glucose[Title/Abstract])) OR (sugar[Title/Abstract])) OR (glycaemia[Title/Abstract])) OR (glycemia[Title/Abstract])) OR ("Blood Glucose"[Mesh]) |
| #7 | (((Triglyceride-glucose index[Title/Abstract]) OR (TyG[Title/Abstract])) OR (triglyceride glucose[Title/Abstract])) OR (triglyceride-glucose[Title/Abstract]) |
| #8 | (((((((Triglycerides[MeSH Major Topic]) OR (Triglycerides[Title/Abstract])) OR (Triacylglycerol[Title/Abstract])) OR (Triacylglycerols[Title/Abstract])) OR (Triglyceride[Title/Abstract])) OR (tryglyceride[Title/Abstract])) OR (triacyl glyceride[Title/Abstract])) AND ((((((Blood Glucose[Title/Abstract]) OR (glucose[Title/Abstract])) OR (sugar[Title/Abstract])) OR (glycaemia[Title/Abstract])) OR (glycemia[Title/Abstract])) OR ("Blood Glucose"[Mesh])) |
| #9 | ((((((((Triglycerides[MeSH Major Topic]) OR (Triglycerides[Title/Abstract])) OR (Triacylglycerol[Title/Abstract])) OR (Triacylglycerols[Title/Abstract])) OR (Triglyceride[Title/Abstract])) OR (tryglyceride[Title/Abstract])) OR (triacyl glyceride[Title/Abstract])) AND ((((((Blood Glucose[Title/Abstract]) OR (glucose[Title/Abstract])) OR (sugar[Title/Abstract])) OR (glycaemia[Title/Abstract])) OR (glycemia[Title/Abstract])) OR ("Blood Glucose"[Mesh]))) OR ((((Triglyceride-glucose index[Title/Abstract]) OR (TyG[Title/Abstract])) OR (triglyceride glucose[Title/Abstract])) OR (triglyceride-glucose[Title/Abstract])) |
| #10 | (((((((((Triglycerides[MeSH Major Topic]) OR (Triglycerides[Title/Abstract])) OR (Triacylglycerol[Title/Abstract])) OR (Triacylglycerols[Title/Abstract])) OR (Triglyceride[Title/Abstract])) OR (tryglyceride[Title/Abstract])) OR (triacyl glyceride[Title/Abstract])) AND ((((((Blood Glucose[Title/Abstract]) OR (glucose[Title/Abstract])) OR (sugar[Title/Abstract])) OR (glycaemia[Title/Abstract])) OR (glycemia[Title/Abstract])) OR ("Blood Glucose"[Mesh]))) OR ((((Triglyceride-glucose index[Title/Abstract]) OR (TyG[Title/Abstract])) OR (triglyceride glucose[Title/Abstract])) OR (triglyceride-glucose[Title/Abstract]))) AND (("Percutaneous Coronary Intervention"[Mesh]) OR (((((Percutaneous Coronary Intervention[Title/Abstract]) OR (PCI[Title/Abstract])) OR (coronary intervention[Title/Abstract])) OR (angioplasty[Title/Abstract])) OR (stent*[Title/Abstract]))) |

**2.Cochrane**

| Search number | Query |
| --- | --- |
| #1 | MeSH descriptor: [Percutaneous Coronary Intervention] explode all trees |
| #2 | (Percutaneous Coronary Intervention):ti,ab,kw OR (PCI):ti,ab,kw OR (coronary intervention):ti,ab,kw OR (angioplasty):ti,ab,kw OR (stent*):ti,ab,kw |
| #3 | #1 or #2 |
| #4 | MeSH descriptor: [Triglycerides] explode all trees |
| #5 | (Triglycerides):ti,ab,kw OR (Triacylglycerol):ti,ab,kw OR (Triacylglycerols):ti,ab,kw OR (Triglyceride):ti,ab,kw OR (tryglyceride):ti,ab,kw |
| #6 | (triacyl glyceride):ti,ab,kw |
| #7 | #4 or #5 or #6 |
| #8 | MeSH descriptor: [Blood Glucose] explode all trees |
| #9 | (Blood Glucose):ti,ab,kw OR (glucose):ti,ab,kw OR (sugar):ti,ab,kw |
| #10 | #8 or #9 |
| #11 | #7 and #10 |
| #12 | (Triglyceride-glucose index):ti,ab,kw OR (TyG):ti,ab,kw OR (triglyceride glucose):ti,ab,kw |
| #13 | #11 or #12 |
| #14 | #3 and #13 |

**3.Embase**

| Search number | Query |
| --- | --- |
| #1 | percutaneous coronary intervention'/exp |
| #2 | percutaneous coronary intervention':ab,ti OR pci:ab,ti OR 'coronary intervention':ab,ti OR angioplasty:ab,ti OR stent*:ab,ti |
| #3 | #1 OR #2 |
| #4 | 'triacylglycerol'/exp |
| #5 | triglycerides:ab,ti OR triacylglycerol:ab,ti OR triacylglycerols:ab,ti OR triglyceride:ab,ti OR tryglyceride:ab,ti |
| #6 | #4 OR #5 |
| #7 | 'glucose blood level'/exp |
| #8 | 'blood glucose':ab,ti OR glucose:ab,ti OR sugar:ab,ti OR glycaemia:ab,ti OR glycemia:ab,ti |
| #9 | #7 OR #8 |
| #10 | #6 AND #9 |
| #11 | 'triglyceride-glucose index'/exp |
| #12 | 'triglyceride-glucose index':ab,ti OR tyg:ab,ti OR 'triglyceride glucose':ab,ti |
| #13 | #11 OR #12 |
| #14 | #10 OR #13 |
| #15 | #3 AND #14 |

**4.Web of science**

| Search number | Query |
| --- | --- |
| #1 | Percutaneous Coronary Intervention (Topic) OR PCI (Topic) OR coronary intervention (Topic) OR angioplasty (Topic) |
| #2 | Triglycerides (Topic) OR Triacylglycerol (Topic) OR Triacylglycerols (Topic) OR Triglyceride (Topic) |
| #3 | Blood Glucose (Topic) OR glucose (Topic) OR sugar (Topic) OR glycaemia (Topic) |
| #4 | Triglyceride-glucose index (Topic) OR TyG (Topic) OR triglyceride glucose (Topic) OR triglyceride-glucose (Topic) |
| #5 | #2 AND #3 |
| #6 | #5 OR #4 |
| #7 | #1 AND #6 |

# 2. statistical methods

The odds ratio is a common measure in medical research of the effect size comparing two groups (treatments or risk factors) in terms of an outcome that is either present or absent. However, the odds ratio is poorly understood.1-3 The relative risk (also called the risk ratio) is more intuitive, but cannot be obtained from case-control studies or (except in rare instances) logistic regressions. The formula for converting an odds ratio to a relative risk is straightforward: Relative risk=odds ratio/(1−p0+(p0×odds ratio))(Where p0 is the baseline risk.)

**Reference:**

1. Grant RL. Converting an odds ratio to a range of plausible relative risks for better communication of research findings. BMJ. 2014 Jan 24;348:f7450.
2. Cummings P. The relative merits of risk ratios and odds ratios. Arch Pediatr Adolesc Med. 2009 May;163(5):438-45.

**Table S1:** Quality assessment of included studies (Newcastle Ottawa Scale)

| **Study** | **Selection** | | | | **Comparability** | **Outcome** | | | **Quality scores** |
| --- | --- | --- | --- | --- | --- | --- | --- | --- | --- |
|  | Representativeness of the exposed cohort | Selection of the nonexposed cohort | Ascertainment of exposure | Demonstration that outcome of interest was not present at start of study | Comparability of cohorts on the basis of the design or analysis | Assessment of outcome | Was follow-up long enough for outcomes to occur | Adequacy of follow up of cohorts |  |
| Zhu 2021 | **＊** | **＊** | **＊** | **＊** | **＊＊** | **＊** | **-** | **＊** | 8 |
| Ferik 2022 | **＊** | **＊** | **＊** | **＊** | **＊＊** | **＊** | **-** | **-** | 7 |
| Guo 2023 | **＊** | **＊** | **＊** | **＊** | **＊** | **＊** | **＊** | **＊** | 8 |
| Huang 2022 | **＊** | **＊** | **＊** | **＊** | **＊** | **＊** | **-** | **-** | 6 |
| Kalyoncuoglu 2021 | **＊** | **＊** | **＊** | **＊** | **＊＊** | **＊** | **＊** | **＊** | 9 |
| Lin2023 | **＊** | **＊** | **-** | **＊** | **＊＊** | **＊** | **-** | **＊** | 7 |
| Yang2022 | **＊** | **＊** | **＊** | **＊** | **＊＊** | **＊** | **＊** | **＊** | 9 |
| Luo2019 | **＊** | **＊** | **-** | **＊** | **＊** | **＊** | **＊** | **＊** | 8 |
| Ma2020 | **＊** | **＊** | **＊** | **＊** | **＊** | **＊** | **＊** | **＊** | 8 |
| Qin2022 | **＊** | **＊** | **＊** | **＊** | **＊＊** | **＊** | **-** | **-** | 7 |
| Sun2023 | **＊** | **＊** | **＊** | **＊** | **＊＊** | **＊** | **＊** | **＊** | 9 |
| Wang2022 | **＊** | **＊** | **＊** | **＊** | **＊** | **＊** | **＊** | **＊** | 8 |
| Xiong2022 | **＊** | **＊** | **＊** | **＊** | **＊＊** | **＊** | **＊** | **＊** | 9 |
| Jie Yang2021 | **-** | **-** | **＊** | **＊** | **＊** | **＊** | **＊** | **-** | 5 |
| Yu2022 | **＊** | **＊** | **＊** | **＊** | **＊＊** | **＊** | **-** | **-** | 7 |
| Zhang2023 | **＊** | **＊** | **＊** | **＊** | **＊＊** | **＊** | **-** | **-** | 7 |
| Zhao2021 | **＊** | **＊** | **＊** | **＊** | **＊** | **＊** | **-** | **-** | 6 |

**Table S2:** Added characteristics of the studies included in the meta-analysis

| No. | First Author | Year | Follow-up time | Study outcomes |
| --- | --- | --- | --- | --- |
| No.1 | Zhu | 2021 | 24months | In-stent restenosis (which was defined as the presence of significant diameter stenosis (≥50%) at the segment inside the stent or involving its 5-mm edges) |
| No.2 | Ferik | 2022 | - | In-stent restenosis (which was defined as the presence of significant diameter stenosis (≥50%) at the segment inside the stent or involving its 5-mm edges) |
| No.3 | Guo | 2023 | 60 months | a composite of PCI complications, including repeat revascularization and in-stent restenosis |
| No.4 | Huang | 2022 | - | Cardiovascular-specific mortality, non-fatal acute MI, and non-fatal stroke |
| No.5 | Kalyoncuoglu | 2021 | 12 months | In-stent restenosis (which was defined as the presence of significant diameter stenosis (≥50%) at the segment inside the stent or involving its 5-mm edges) |
| No.6 | Lin | 2023 | 22 months | the composite of overall death, nonfatal MI, and unplanned revascularization. |
| No.7 | Yang | 2022 | 35 months | new-onset atrial fibrillation |
| No.8 | Luo | 2019 | 12 months | All-cause death, target vessel revascularization, MI during follow-up, unstable angina pectoris requiring hospitalization, heart failure, stroke or transient cerebral ischaemia |
| No.9 | Ma | 2020 | 30 months | the composite of overall death, non-fatal stroke, non-fatal myocardial infarction (MI),or unplanned repeat revascularization |
| No.10 | Qin | 2022 | 23 months | All-cause death, non-fatal MI, target vessel reconstruction, malignant arrhythmia, angina pectoris requiring hospitalization, and acute heart failure |
| No.11 | Sun | 2023 | 36 months | All-cause mortality, non-fatal MI, and any revascularization |
| No.12 | Wang | 2022 | 2 months | a composite of all-cause death, nonfatal myocardial infarction (MI), unplanned revascularization, and ischemic stroke |
| No.13 | Xiong | 2022 | 30 months | A composite of all-cause death, non-fatal MI, and unplanned revascularization during follow-up |
| No.14 | Yang jie | 2021 | 24 months | Primary endpoint :major adverse cardiovascular and cerebrovascular events (MACCE), including all-cause mortality, nonfatal MI, nonfatal stroke, and target vessel revascularization (TVR). The secondary outcomes: MACE (a composite of cardiac death, nonfatal MI, all-cause death, nonfatal stroke, and TVR.) |
| No.15 | Yu | 2022 | - | Post-PCI reduction of quantitative flow ratio(QFR) |
| No.16 | Zhang | 2023 | 66 months | the composite of cardiac rehospitalization (admission because of angina or heart failure), ischemia-driven revascularization, non-fatal ischemic stroke, non-fatal myocardial infarction (MI), cardiac death, and all-cause death |
| No.17 | Zhao | 2021 | 24 months | All-cause death, non-fatal MI, non-fatal ischemic stroke, and ischemia-driven revascularization |


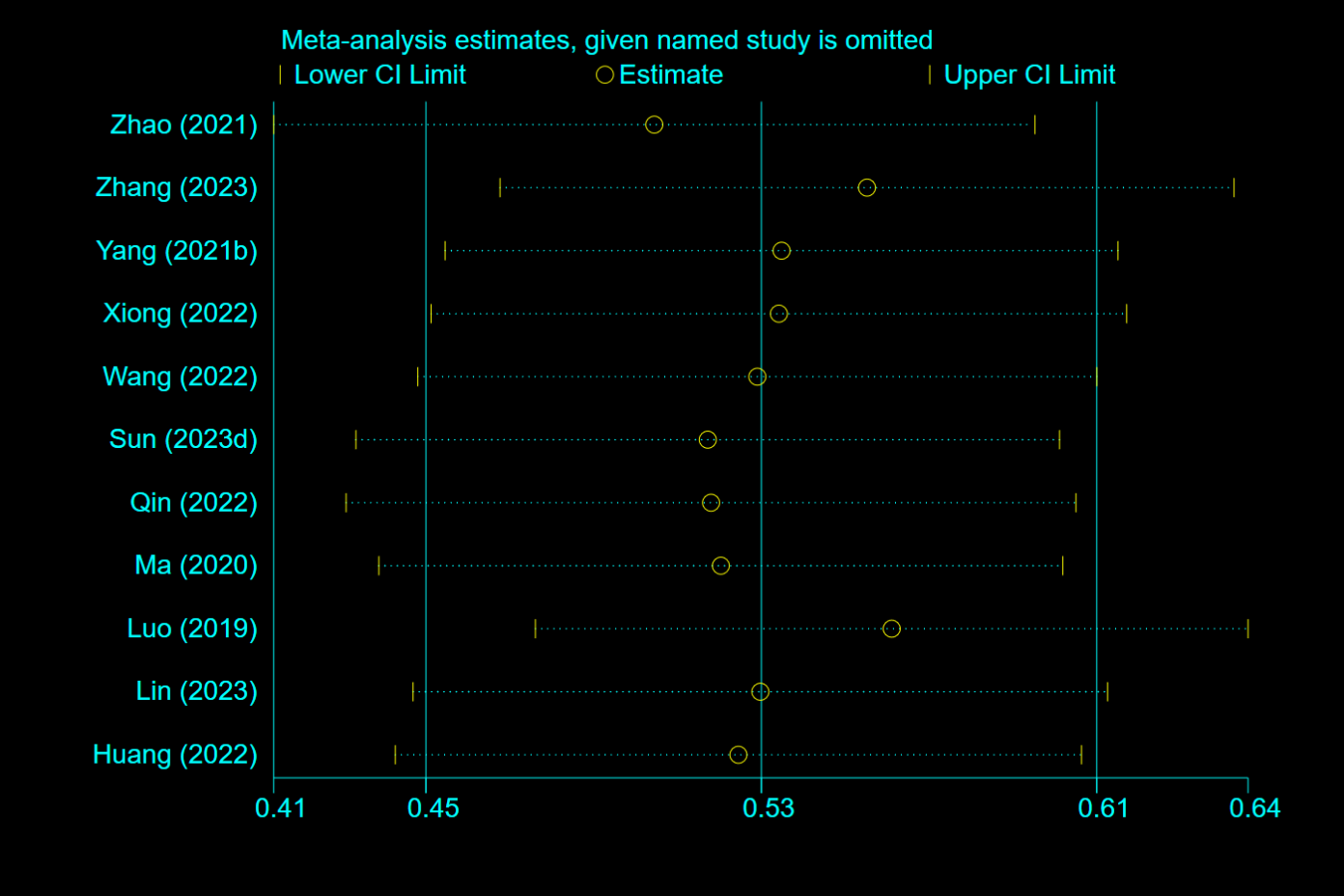


**Figure S1:** Publication bias analysis for primary endpoint

**
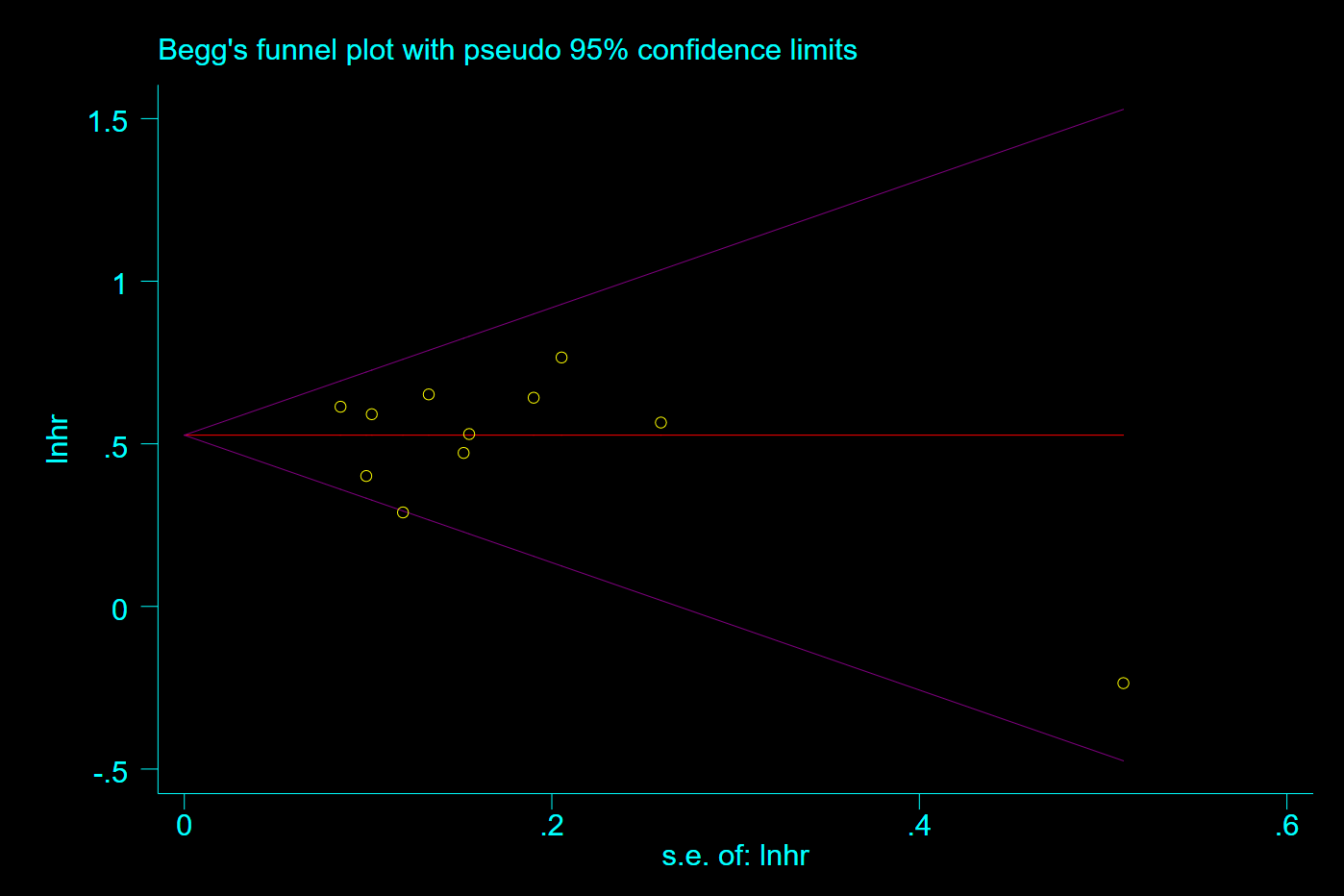
**

**Figure S2:** Begg's test for primary endpoint

**the study by Sun et al. was reported as a single study in the meta-analysis to prevent over-representation in the meta-analysis. The internal meta-analysis for this study was presented as below.**

**Figure S3（A）:**

The meta-analysis of the incidence of MACE in post-PCI patients (categorized variables included. Sun (2023a), All-cause mortality; Sun (2023b), Non-fatal MI; Sun (2023c),revascularization; Sun (2023d), MACE; Sun (2023e), MACE in patients with DM; Sun (2023f), MACE in patients without DM.)


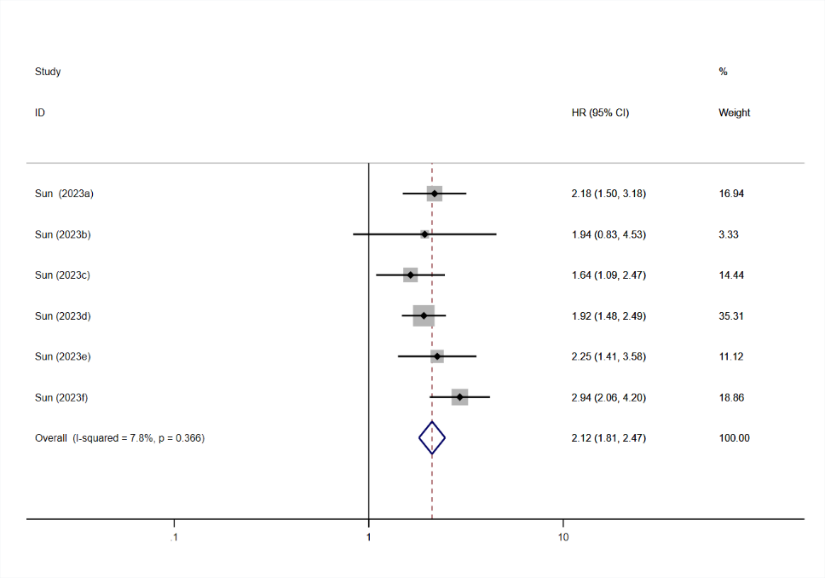


**Figure S3（B）:**

The meta-analysis of the incidence of MACE in post-PCI patients (continuous variables included. Sun (2023a), MACE; Sun (2023b), All-cause mortality; Sun (2023c), Non-fatal MI; Sun (2023d),


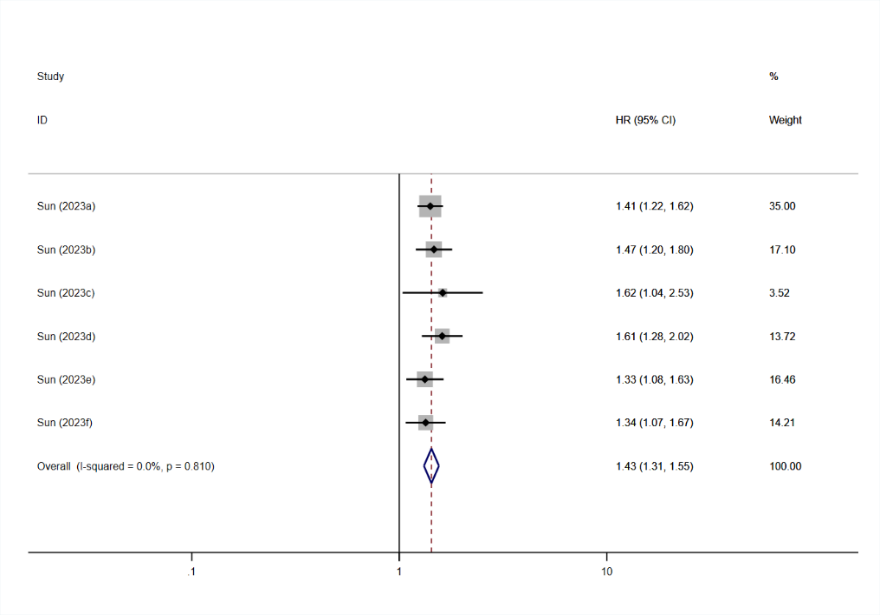


MACE in patients with DM; Sun (2023e), MACE in patients without DM; Sun (2023f), revascularization.)

**Figure S4** Subgroup analysis based on diabetes(continuous variables included. Sun (2023a), risk of MACE in patients with DM; Sun (2023b), risk of MACE in patients without DM)


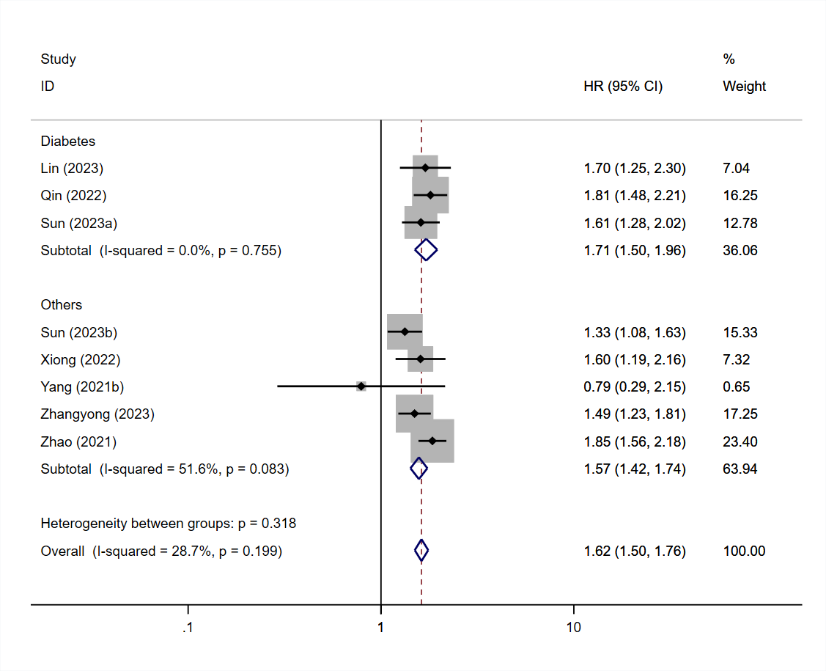


**Figure S5** Subgroup analysis based on duration of follow-up (continuous variables included.)
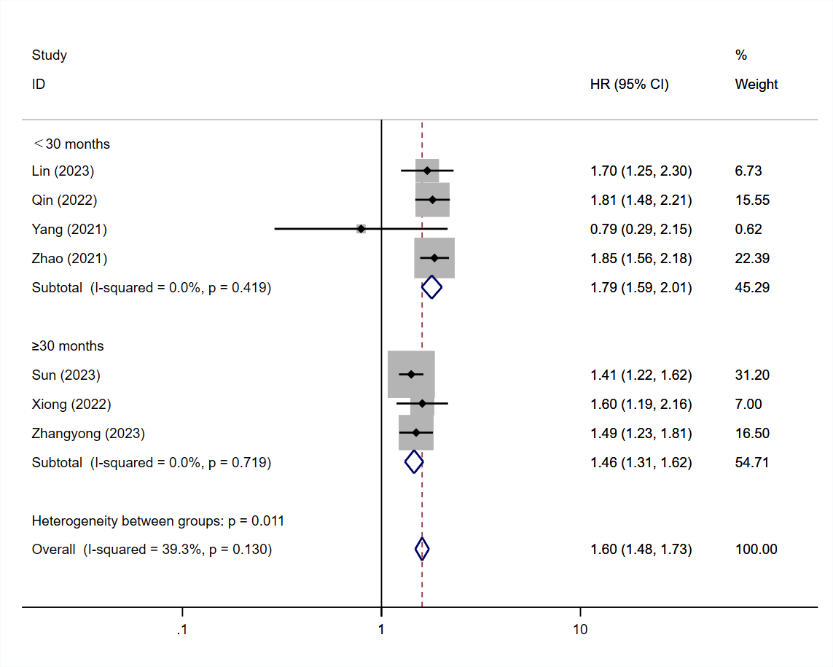

Supplement: Supplementary file 1 [file Datasheet1.docx]
